# A phase II study of perioperative pembrolizumab plus mFOLFOX in patients with potentially resectable esophagus, gastroesophageal junction (GEJ), and stomach adenocarcinoma

**DOI:** 10.1002/cam4.6263

**Published:** 2023-06-16

**Authors:** Weijing Sun, Nirmal Veeramachaneni, Raed Al‐Rajabi, Rashna Madan, Anup Kasi, Mazin Al‐Kasspooles, Joaquina Baranda, Anwaar Saeed, Milind A. Phadnis, Andrew K. Godwin, Mojtaba Olyaee, Natalie Streeter, Alykhan Nagji, Junqiang Dai, Stephen Williamson

**Affiliations:** ^1^ Division of Medical Oncology, Department of Internal Medicine University of Kansas Medical Center Kansas City Kansas USA; ^2^ University of Kansas Cancer Center Kansas City Kansas USA; ^3^ Cardiothoracic Surgery Division, Department of Surgery Saint Louis University School of Medicine Saint Louis Missouri USA; ^4^ Department of Pathology University of Kansas Medical Center Kansas City Kansas USA; ^5^ Division of Surgical Oncology, Department of Surgery University of Kansas Medical Center Kansas City Kansas USA; ^6^ Hematology‐Oncology Division, Department of Medicine University of Pittsburgh Medical Center Pittsburgh Pennsylvania USA; ^7^ Department of Biostatistics and Data Science University of Kansas Medical Center Kansas City Kansas USA; ^8^ Division of Gastroenterology, Department of Internal Medicine University of Kansas Medical Center Kansas City Kansas USA; ^9^ Department of Cardiothoracic Surgery University of Kansas Medical Center Kansas City Kansas USA

**Keywords:** adenocarcinoma, CD8, esophageal gastric, GEJ, immune checkpoint inhibitor (ICI), pathological response (ypRR), PD‐L1, pembrolizumab, perioperative, tumor regression score (TRS)

## Abstract

**Background:**

Perioperative chemotherapy/chemoradiation is standard in esophageal/gastric/gastroesophageal junction (GEJ) adenocarcinoma, immune checkpoint inhibitors (ICI) effect in setting of metastatic and postoperatively. This study is to assess ICI + chemotherapy perioperatively.

**Methods:**

Patients with locally advanced (T1N1‐3M0 or T2‐3NanyM0) potentially resectable esophageal/gastric/GEJ adenocarcinoma by PET/EUS/CT and staging‐laparoscopy, were treated preoperative 4 cycles mFOLFOX6 (Oxaliplatin 85 mg/m^2^/Leucovorin 400 mg/m^2^/5‐FU bolus 400 mg/m^2^ then infusion 2400 mg/m^2^ for 46 h q2weeks) and 3 cycles pembrolizumab (200 mg q3week). Those without distal disease post‐neoadjuvant and eligible for resection underwent surgery. Postoperative treatment was initiated at 4–8 weeks with 4 cycles mFOLFOX and 12 cycles pembrolizumab. The primary objective is pathological response (ypRR with tumor regression score, TRS ≤2). The expression of ICI‐related markers PD‐L1 (CPS), CD8, and CD20 were analyzed before and after preoperative therapy.

**Results:**

Thirty‐seven patients completed the preoperative treatment. Twenty‐nine patients had curative R0 resection. 6/29 (21%; 95% CI: 0.08–0.40) achieved ypCR with TRS 0 in resected patients. 26/29 (90%; 95% CI: 0.73–0.98) had ypRR with TRS ≤2. Twenty‐six patients finished adjuvant therapy with a median 36.3‐month follow‐up. Three patients had recurrence/metastatic disease (at 9‐, 10‐, 22 months enrollment) with one dead at 23 months, and two are still alive at 28 and 36.5 months. The remaining (23/26) are free of disease with 3 years DFS of 88.5% and 3 years OS of 92.3%. There were no unexpected toxicities. Preoperative ICI + chemotherapy enhanced immune responses significantly with increasing expression of PD‐L1 (CPS ≥10, *p* = 0.0078) and CD8 (>5%, *p* = 0.0059).

**Conclusions:**

The perioperative pembrolizumab and mFOLFOX combination in resectable esophageal/gastric/GEJ adenocarcinoma is very effective with 90% ypRR, 21% ypCR, and impressive long‐time survival benefits.

## Graphical abstract

This phase II study assessed the combination of the immune checkpoint inhibitor, pembrolizumab, and chemotherapy mFLOFOX perioperatively in locally advanced esophageal/gastric/GEJ adenocarcinoma. The results showed impressive results of 90% ypRR, 21% ypCR, and impressive long‐time survival benefits (88.5% of 3 years, DFS of 88.5%, and 3 years OS of 92.3%) with demonstrating perioperative therapy increasing PD‐L1 (CPR ≥10, *p* = 0.0078) and CD8 (>5%, *p* = 0.0059) expression significantly.

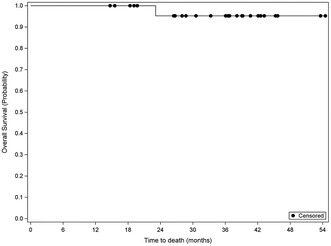

## 1 INTRODUCTION

Cancers of the upper gastrointestinal tract including esophageal, gastric, and gastroesophageal junction (GEJ) cancers are a group of highly aggressive malignancies and a major public health problem globally. Approximately 604,000 new cases with 544,000 deaths of esophageal cancer and over 1 million new cases with 769,000 deaths of gastric cancer are diagnosed in 2020.^1^ In the United States alone, 20,640 new cases of esophageal and 26,380 cases of stomach cancer are estimated to be diagnosed in 2022, with approximately 16,410 and 11,090 deaths, respectively.^2^ While the incidence of esophageal squamous cell carcinoma is declining, the rates of adenocarcinoma are rising rapidly in most populations, which represents roughly two‐thirds of esophageal cancer cases in high‐income countries, with excess body weight, gastroesophageal reflux disease, and Barrett's esophagus among the key risk factors.^3^ Emerging evidence suggests that the etiology for cardia gastric cancer, resembles that characteristic of esophageal adenocarcinoma.^4^ Despite the evolution in the management of locoregional disease with multimodality treatment strategies, esophageal (including GEJ) and gastric cancers continue to remain among the most lethal malignancies with 5‐year survival rates reaching 19.9% and 32%, respectively.^2^ Surgical resection is the potentially curative intervention for locally advanced adenocarcinoma of esophagus, GEJ, and stomach. Studies have confirmed the benefits of perioperative treatment including neoadjuvant and adjuvant chemotherapy or chemoradiation.^5^, ^6^, ^7^, ^8^ However, there have been debates regarding precision treatment option(s) and the lack of a universally accepted standard because of heterogeneity of disease histology (squamous cell carcinoma vs. adenocarcinoma); different chemotherapy regimens, sequences, and with and without radiation. More effective and less toxic treatment approaches are a great and unmet need. Adjuvant chemotherapy showed some survival benefit in patients with gastric adenocarcinoma after curative surgery.^7^ The benefit of neoadjuvant chemoradiation has been proven with improvement of pathologic response rate and resectability with more benefit in squamous cell carcinoma than adenocarcinoma.^6^ However, the risk of recurrence remains high, especially among those patients who have not achieved a pathological complete response.^9^, ^10^ The tolerability of perioperative cytotoxic chemotherapy regimens of ECF (epirubicin, cisplatin, and 5‐flurourcil) and FLOT (5‐flurouracil, leucovorin, oxaliplatin, docetaxel) has been a main issue for compliance. Although FLOT is relatively less toxic than ECF with 90% of the patients completing all cycles of allocated preoperative chemotherapy, only 60% started the allocated postoperative chemotherapy. Consequently, less than half of the patients (46%) completed all the allocated cycles. The FOLFOX regimen has been well accepted in treatment of upper gastrointestinal tract cancers with less toxicity.^11^ The efficacy of immune checkpoint inhibitors combinations with or without chemotherapy in esophageal, GEJ, and gastric cancer have been shown in several phase II studies in the advanced/metastatic disease setting.^12^, ^13^ Recent data demonstrated the benefit of an immune checkpoint inhibitor (ICI) as a single agent at the adjuvant setting in esophageal cancer patients who had preoperative chemoradiation.^14^ The efficacy of pembrolizumab, ICI, in combination with chemotherapy needs to be assessed and confirmed at the perioperative setting.

This single arm phase II trial is aimed to evaluate efficacy and safety of pembrolizumab, in combination with mFOLFOX at the perioperative (both pre‐ and post‐operative) setting in patients with potentially resectable adenocarcinoma of distal esophagus, GEJ, and stomach. The study is also designed to analyze the expression of ICI treatment related markers PD‐L1 (CPS), CD8, and CD20 in the real time before and after preoperative therapy.

## 2 METHODS

### 2.1 Study design

The enrolled patients were at least 18 years old with newly diagnosed locally advanced (T1N1‐3M0 or T2‐3NanyM0), potentially resectable, and histologically or cytologically‐confirmed adenocarcinoma of distal esophagus, GEJ, and stomach. All participants were evaluated with CT chest/abdomen/pelvis (C/A/P), EUS, PET scan, and laparoscopy exams, and showed no evidence of distant metastases. All participants had to be eligible and physically fit to undergo curative surgical resection with ECOG performance status of 0 or 1. Participants had to meet the following laboratory parameters: hemoglobin ≥9/mm^3^, ANC count ≥1500/mm^3^, platelets ≥100,000/mm^3^, adequate hepatic function with bilirubin <2× upper limit of normal (ULN), and AST and ALT ≤2.5 × ULN. All participants were required to understand, and sign written informed consent.

### 2.2 Procedures and treatment

The patients received preoperative treatment with the combination of mFOLFOX (oxaliplatin 85 mg/m^2^, leucovorin 400 mg/m^2^, 5‐FU bolus 400 mg/m^2^, and 5‐FU 2400 mg/m^2^ infusion over 46 h) every 2 weeks for 4 doses (on days 1, 15, 29, 43) and pembrolizumab (200 mg IV) every 3 weeks for 3 doses (on days 1, 22, 43).

Repeat PET and CT C/A/P were obtained approximately 2–3 weeks after completion of neoadjuvant combination therapy. Those patients with no evidence of metastatic disease on PET & CT C/A/P and physically fit for operation underwent potentially curative surgical resection 4–8 weeks after completion of neoadjuvant combination therapy. If there was evidence of metastatic disease on PET &CT C/A/P, or physically not fit for operation, the participant came off the study (i.e., did not undergo surgical resection and adjuvant therapy) and managed with the standard care.

Postoperative adjuvant treatment (started 6–8 weeks after surgery) consisted of mFOLFOX every 2 weeks for additional 4 doses plus pembrolizumab every 3 weeks for additional 12 doses (total 1 year of therapy). Patients continued to receive treatment until either one of the following occurred: completion of adjuvant therapy, development of radiographically confirmed progression, the participant withdrew consent, intercurrent illness that prevented further administration of treatment, or the sponsor‐investigator decided to withdraw the participant. Efficacy outcomes during the adjuvant chemotherapy phase were determined by radiologic measurements through CT C/A/P. Assessment of response were performed every 3 months for the first year and as per the standard institutional guidelines thereafter.

Tissue analysis: Pretreatment biopsies and surgical specimens were analyzed for the immunohistochemical expression of ICI treatment related markers: PD‐L1, CD8, and CD20 before and after preoperative pembrolizumab + mFOLFOX therapy.

Formalin‐fixed paraffin‐embedded 4‐μm thick whole tissue sections were utilized for immunohistochemical staining. Staining for PD‐L1 (clone 22C3) was performed in the standardized manner using the EnVision FLEX visualization system on the Autostainer Link 48 (all: Dako/Agilent Tecnologies, Inc.). Staining for CD8 (clone C8/144B, Biocare Medical) at 1:150 dilution following antigen retrieval using DIVA Decloaker (DV2004, Biocare Medical), and for CD20 at 1:100 dilution following antigen retrieval using Target Retrieval Solution, Citrate pH 6.1 (10x) (Dako/Agilent Tecnologies, Inc.) were run on the Intellipath FLX autostainer (Biocare Medical) with the polymer refine detection Anti‐Mouse Envision + System HRP (K400, Dako/Agilent Tecnologies, Inc.).

PD‐L1 staining was scored utilizing the standardized Combined Positive Score (CPS). CD8 and CD20 were each assessed as the percentage of all cells in the tumor area.

### 2.3 Endpoints

The primary objectives were pathological response rate (ypRR with tumor regression score, TRS ≤2), and safety/tolerability of the combination. The tumor regression score (TRS) was based on pathology review, and recorded for each patient according to the following categories: 0 (complete response: no viable cancer cells), 1 (near complete response: single cells or rare small groups of cancer cells), 2 (partial response: residual cancer showing evident tumor regression, but more than single cells or rare small groups of cancer cells), or 3 (poor or no response: extensive residual cancer and no evident tumor regression). The secondary objectives were to evaluate the antitumor activity of the combination as determined by objective response rate (ORR), disease‐free survival (DFS), and overall survival (OS). This single institutional study was fully approved by the institutional review board (IRB) of the University Kansas Medical Center with Trial registration – ClinicalTrials.gov Identifier: NCT03488667.

### 2.4 Statistical analysis

#### 2.4.1 Sample size justification

The sample size calculation was based on the research hypothesis that the response rate for the combination treatment will be greater than 30% (as suggested by historical data). Using a conservative effect size indicative of a 20% improvement in the response rate for the combination therapy, a sample size of 40 evaluable patients was initially calculated to obtain 81% power using a one‐sided *z*‐test of proportions (normal approximation with continuity correction) with a type I error of 5%.

#### 2.4.2 Interim analysis

Interim analyses involved the implementation of study stopping rules related to the joint monitoring of efficacy and toxicity using the Bayesian sequential monitoring design of Simon, Thall and Etsey. The DLTs for early stopping were defined as non‐hematologic AEs of grade ≥3 or hematologic toxicity of grade ≥4 (evaluated by CTCAE 4.0). We consider the combination treatment as promising and worthy of future investigation in a larger trial if the response rate is ≥50% and the SAE rate is ≤30%. Study stopping boundaries were calculated based on posterior probabilities of ypRR or SAE rates exceeding prespecified threshold values (representing the expected performance of standard treatment). A run‐in period of 6 patients was used and stopping rules were evaluated every 5 patients thereafter (until a maximum of 36 patients). Based on the observed response rates and SAEs, the study was not halted for early evidence of futility or toxicity and continued till its planned end.

#### 2.4.3 Final analysis

Summary statistics were reported for the patient characteristics. Categorical variables were reported using frequency counts and proportions whereas continuous variables were reported using the median and range. The primary outcome of ypRR was reported as the proportion of subjects with TRS ≤2 and its corresponding 95% exact Clopper‐Pearson interval. A one‐sided *z*‐test of proportions (normal approximation) was used to compare this observed estimate to the hypothesized value of 30%. The secondary outcome of AE related toxicity was reported using its category and grade. Likewise, the secondary outcome of ypCR and ORR at 12 weeks were reported using exact Clopper–Pearson 95% confidence intervals. Kaplan–Meier survival estimates were plotted for DFS and OS. The SAS 9.4 statistical software was used to perform all analyses and all tests were carried out at the 5% significance level. A McNemar's test was used to test the association between preoperative ICI + chemotherapy and increasing expression of PDL1 (CPS ≥10), CD8 (≥5%), and CD 20 (≥5%).

## 3 RESULTS

From 7/11/2018 to 11/21/2021, 37 patients were enrolled in the study and finished preoperative treatment as planned with mean age of 65 year's old (range 44–86 year's old) (Table 1). There were 30 males and 7 females. All were adenocarcinoma by histopathology with the differentiation as: 4 well, 11 moderately, 1 moderately to poorly, 19 poorly. All patients were staged clinically as locally advanced diseases: 1 as T_1_ (N_1_); 11 as T_2_, 24 as T_3_, 1 as T_4_; and 12 N_0_ (5 T_2_, 4 T_3,_ & 1 T_4_), 12 as N_1_, 10 as N_2_, and 3 as N_3_. The location of primary disease as follows: 14 at distal esophagus, 11 at the GEJ, and 12 at the stomach.

**TABLE 1:** Patient characteristics: (*N* = 37).

Age: median (range)	65 (44–86)
Gender: male/female	30/7
Location	
Distal esophagus	14
GEJ	11
Gastric	12
T stage	
T1	1
T2	11
T3	24
T4	1
N stage	
N0	12
N1	12
N2	10
N3	3
Differentiation	
Well	4
Moderate	11
Moderate to poorly	1
Poorly	19

In those 37 patients who finished preoperative treatment evaluation, 8 patients did not have surgical resection. Two subjects non‐operable from medical reasons (one with hepatic cirrhosis and one because of physical unfitness) and six due to progression or non‐resectable disease. Ninety‐one patients have disease progression by the planned image assessment after neoadjuvant therapy, 1 found retroperitoneal LN; 1 with micro‐metastatic lesion in the surface of the liver which was not seen in the screening laparoscopy; 1 with peritoneal disease; 2 with aorta involvement and high risk for surgical resection. Twenty‐nine patients had curative intended operations, and all had R0 resection. Six of 29 (21%; 95% CI: 0.08–0.40) achieved ypCR with TRS of 0 in the primary cancer. All except three patients (26/29, 90%, 95% CI: 0.73–0.98) had shown pathologic response to the treatment with TRS ≤2. This ypRR rate of 90% was significantly higher than the hypothesized rate of 30% as assessed by a *z*‐test of proportions (*p* value < 0.0001). Though the initial sample size was calculated as *N* = 40, our ability to enroll *N* = 37 did not compromise the statistical power of the study.

There were three patients who did not receive postoperative adjuvant treatment for various reasons: one patient's final pathology of the surgical specimen showed mixed adenocarcinoma with neuroendocrine tumor; one patient did not recover from surgery; and one patient suffered sudden death post operation which was not treatment related.

**FIGURE 1:**
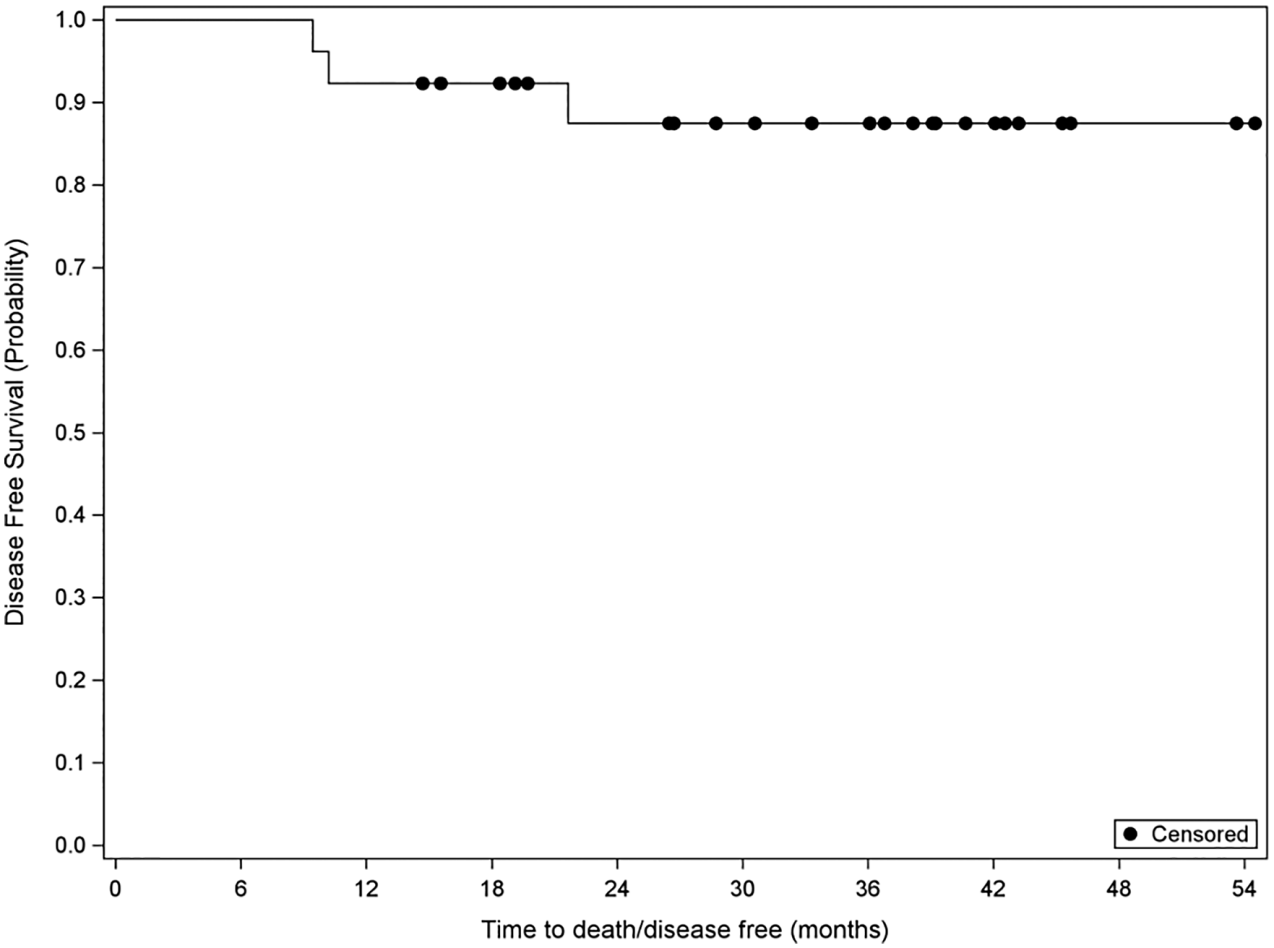
Disease Free Survival Median follow‐up of 36.3 months (12.7–52.2 months). Date of all 26 patients with finished postoperative adjuvant therapy, with the median follow‐up of 36.3 months (12.7–52.2 months).

**FIGURE 2:**
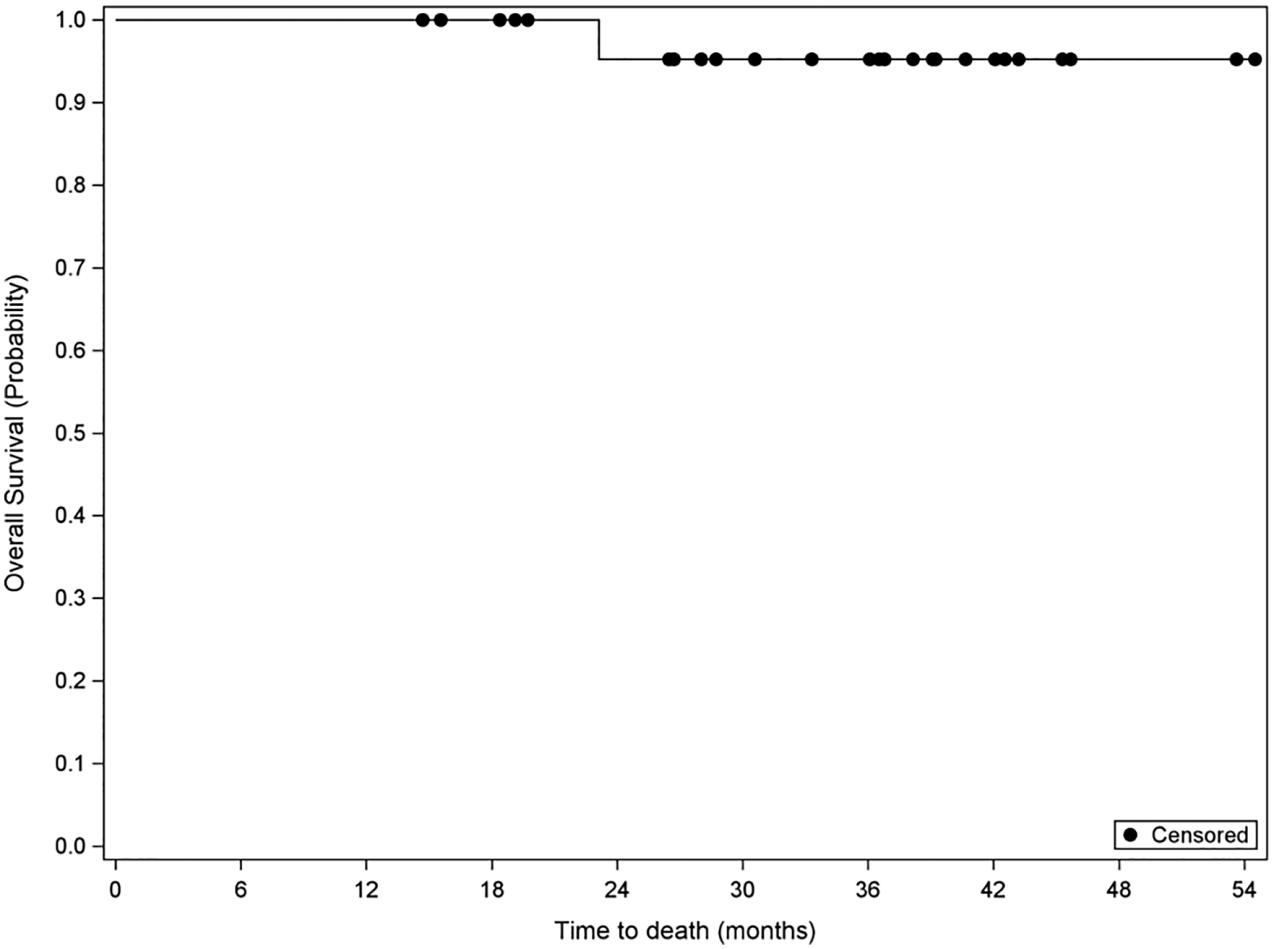
Overall Survival Median follow‐up of 36.3 months (12.7–52.2 months). Date of all 26 patients with finished postoperative adjuvant therapy; the median follow‐up with the median follow‐up of 36.3 months (12.7–52.2 months).

**FIGURE 3:**
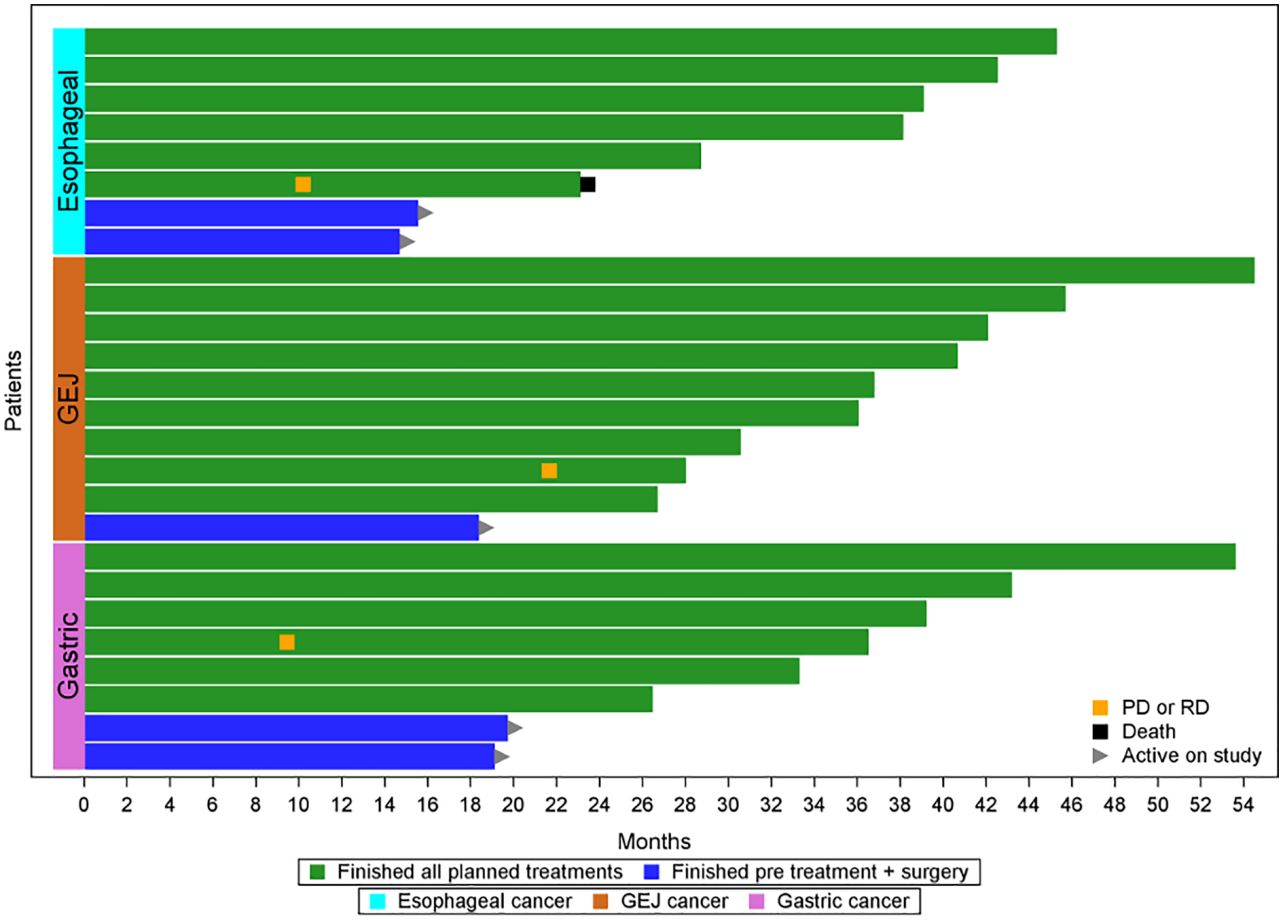
Efficacy based on the tumor location. Date of all 26 patients with finished postoperative adjuvant therapy, the median follow‐up with the median follow‐up of 36.3 months (12.7–52.2 months).

Twenty‐six patients finished adjuvant therapy with a median follow‐up of 36.3 months (12.7–52.5 months). Three had recurrence/metastatic disease (at 9‐, 10‐, and 22 months enrollment) with one dead at 23 months, and the others are still alive at 28 and 36.5 months. In those three patients with recurrent and metastatic diseases, one had liver metastatic lesion at 10 months of enrollment, one had a splenic lesion at 9.5 months of enrollment, and one had skin metastatic lesions at 22 months from the enrollment. The remaining patients (23/26) are all free of disease with 3 years DFS of 88.5% and 3 years OS of 92.3%, the median follow‐up is 36.3 months (12.7–52.2 months) (Figures 1 and 2). There were no efficacy differences shown based on the locations (esophagus, GEJ, and stomach) of the adenocarcinoma. (Figure 3).

The toxicity data showed that the main toxicities from the combination were chemotherapy regimen mFOLFOX related: nausea, vomiting, diarrhea, peripheral sensory neuropathy, fatigue, mucositis, neutropenia. Arthralgia, pneumonitis, adrenal insufficiency, hepatitis, and colitis were observed and considered to be related to ICI, pembrolizumab, with one case of G3 of each. There were no unexpected toxicities. G3/4 toxicities were reported in 21 of all 37 treated patients (Table 2).

**TABLE 2:** Toxicity of the combination treatment (in all patients, *N* = 37).

Toxicity	All grade no. (%)	Grade no. (%)
Nausea	15 (41)	3 (8)
Vomiting	10 (27)	3 (8)
Diarrhea	12 (32)	1 (3)
Abdominal pain	11 (30)	2 (5)
Anorexia	8 (22)	2 (5)
Constipation	8 (22)	1 (3)
Weight loss	7 (19)	1 (3)
Hypertension	4 (11)	1 (3)
Peripheral sensory neuropathy	15 (41)	0 (0)
Fatigue	13 (35)	0 (0)
Mucositis	8 (22)	0 (0)
Anxiety	5 (14)	0 (0)
Dizziness	4 (11)	0 (0)
Generalized muscle weakness	3 (8)	0 (0)
Dyspnea	3 (8)	2 (5)
Esophagitis	1 (3)	1 (3)
Pneumonia	1 (3)	1 (3)
Atelectasis	1 (3)	1 (3)
Thromboembolic event	1 (3)	1 (3)
Rash maculopapular	1 (3)	0 (0)
Rash acneiform	1 (3)	0 (0)
Neutropenia	12 (32)	8 (22)
Thromboembolic event	1 (3)	1 (3)
Anemia	1 (3)	0 (0)
Hypokalemia	5 (14)	1 (3)
Hyperglycemia	2 (5)	1 (3)
Hypophosphatemia	1 (3)	1 (3)
Arthralgia	5 (16)	1 (3)
Pneumonitis	2 (5)	1 (3)
Adrenal insufficiency	1 (3)	1 (3)
Liver function abnormality	1 (3)	1 (3)
Colitis	1 (3)	1 (3)
Sepsis (post operation)	3 (8)	3 (8)
Atrial fibrillation	1 (3)	1 (3)
Procedural complications	1 (3)	1 (3)

All patients (100%) completed preoperative treatment (68% completed without dose modification, and the 32% with mild (<10%) dose modification based on the protocol). All patients qualified for postoperative treatment completed the combination therapy (58% without dose modification, and 42% with mild (<10%) dose modification based on the protocol).

Analyses of the expression of ICI treatment related markers PD‐L1 (CPS), CD8, and CD20 before and after preoperative therapy were performed in those patients who had surgery resection. Those patients achieved ypCR were excluded since no tumor was seen in their surgical specimens. The analyses demonstrated a significant increase in PD‐L1 (CPS ≥10) in the surgical specimens after the preoperative treatment over the biopsy specimens (*p* = 0.0078). CD8 expression (>5%) also showed significant increase in the surgical specimens after the preoperative treatment compared to the biopsy specimens (*p* = 0.0059). However, there was no difference in the CD20 expression before and after preoperative therapy.

## 4 DISCUSSION

Surgical resection reaction is the only potentially curative intervention for locally advanced adenocarcinoma of esophagus, gastroesophageal junction (GEJ), and stomach. Results from various studies have demonstrated the benefits of perioperative treatment including neoadjuvant and adjuvant chemotherapy or chemoradiation; however, there is lack of universally accepted standard based on several factors: heterogeneity of histopathological and biological disease characteristics, local‐regional versus systemic benefits from chemoradiation, and tolerability and toxicity of systemic cytotoxic chemotherapy regimens.

The data of the neoadjuvant chemoradiation in esophageal and GEJ cancer (Cross Trial) showed that the histopathologic type of adenocarcinoma (HR 0.741, 95% CI 0.536–2.024, *p* = 0.07) did not gain as much benefit as that of squamous cell carcinoma (HR 0.422, 95% CI 0.226–0.788, *p* = 0.007) from the treatment.^6^ The 10 years follow‐up of the Cross trial exhibited that the absolute survival benefits (38% vs. 25%) from the neoadjuvant chemoradiation are mainly contributed by local‐reginal effects of the therapy (HR, 0.43; 95% CI, 0.26–0.72), without clear differences in distance recurrence (HR, 0.76; 95% CI, 0.52–1.13), which is likely because of no postoperative adjuvant therapy.^15^ The most recently published study of adding nivolumab, ICI, as adjuvant therapy in the population of patients who had neoadjuvant chemoradiation has demonstrated the significance of postoperative therapy to clearing the micro‐metastatic disease after potential curative surgery and declared the role of ICI at the adjuvant setting.

The perioperative triple agent combination showed evidence of survival benefit from original MAGIC trial of ECF (epirubicin, cisplatin, and 5‐flurourcil) compared with surgery alone (5‐year survival rates, 36% vs. 23%),^5^ however, the tolerability was the major challenge with only 46% participates finishing the planned chemotherapy treatment because of toxicity. Compared to ECF/ECX (epirubicin, cisplatin, and capecitabine), FLOT (5‐flurouracil, leucovorin, oxaliplatin, docetaxel) showed improved survival benefit with median overall survival, 50 months versus 35 months. However, the serious adverse events were the same as ECF/ECX with less grade 3 or 4 non‐hematological toxicities but more grade 3 or 4 neutropenia and infection.^8^ The toxicity of FOLFOX or CAPOX is much less than either ECF/ECX or FLOT.^7^, ^11^ Efforts have been undertaken for decreasing cytotoxic chemotherapy in the perioperative setting, with FOLFOX being the most used alternative regimen in clinical practice. However, since FLOT has been the regimen of the standard of care (SoC) in the Europe, the worry of compromising efficacy with FOLFOX at that setting is still a concern. A randomized phase IIb study of AIO German Gastric Cancer Group and Swiss SAKK (DANTE Trial) compared FLOT + atezolizumab versus FLOT in patients with resectable esophagogastric adenomarcinoma (20), the interim result showed “beneficial effects of atezolizumab combined with FLOT vs. FLOT alone on pathological stage and pathological regression that seem to be more pronounced with higher PD‐L1 expression”. However, the presentation at the ASCO 2022 annual meeting showed high grade 3–4 toxicities in both arms (90% in FLOT + Atezolizumab vs. 85% in the FLOT arm) and 5% grade 5 toxicity in both arms, those were mainly from FLOT combination.

The significance of pathologic response from the perioperative treatment and its impact on the survival outcome has been established in several studies.^7^, ^9^, ^10^, ^16^ This study design of perioperative regimen with combination of pembrolizumab and mFOLFOX in patients with potentially resectable adenocarcinoma of distal esophagus, GEJ, and stomach is addressing all concerns as discussed above, and trying to achieve the best outcomes including pathological response and survival benefit while minimizing the treatment‐related toxicity.

This is the first phase II study demonstrating the benefits of the combination of ICI, pembrolizumab, with the cytotoxic chemotherapy regimen, mFOLFOX, at the perioperative setting in patients with locally advanced adenocarcinoma of distal esophagus, GEJ, and stomach.

The study population represents the clinical setting with median age of 65 (range 44–86) with more male (30/37) patients. Majority of patients had high degree of locally advanced disease with 25/37 with ≥T_3_, and 25/37 ≥N_1_ diseases. All patients had adenocarcinoma which eliminate the histopathologic heterogeneity in the study population, and the study showed the no differences in efficacy based on the tumor location (esophagus, GEJ, and stomach).

The study met its end points with 21% ypCR (TRS 0, 95% CI: 0.08–0.40) in the primary lesions, and 90% ypRR (TRS ≤2, 95% CI: 0.73–0.98) for patients who underwent curative surgical resection. With the median follow‐up of 36.3 months (12.7–52.2 months), three patients had recurrent/metastatic disease, and one died, the remaining patients (23/26) are all free of disease. The 3 years DFS is 88.5% and 3 years OS is 92.3%. The toxicity data is also encouraging with no unexpected toxicities, and the combination regimen was fairly tolerable in all eligible patients. There were no patients dropping out from the study in either preoperative or postoperative setting because of toxicity. All eligible patients finished planned combination therapy. The mild dose modifications were reported in 32% of patients during the neoadjuvant period; and 42% during the adjuvant period.

As a part of the study design, we analyzed the expression of ICI treatment related markers PD‐L1, CD8, and CD20 in the real time before and after preoperative therapy. Several large phase II studies indicated that the PD‐L1 expression level is significant for the anti‐PD‐1/PD‐L1 efficacy at the metastatic setting (12,13). A cohort study with 179 NSCLC quantitative immunofluorescence panels to determine the association of major TILs subpopulations, CD8+, CD4+, CD20+ B cells; PD‐1, LAG3, and TIM‐3T cells with outcomes. The result showed higher density of stromal CD8+ cells was associated the longer survival significantly, and more effect in PD‐L1 cases.^17^ The results of this study clearly demonstrated that the preoperative pembrolizimab + FOLFOX enhances the immune response with increased expression of PD‐L1 (PD‐L1 CPS ≥10, *p* = 0.0078) and CD8 (CD8 ≥5%, *p* = 0.0059), augmented the anti‐tumor efficacy of the combination treatment, also enhances adjuvant treatment benefits and thereafter the overall DFS and OS. Therefore, pembrolizumab adding into the standard preoperative chemotherapy should be considered as the standard with further confirmative studies. These data consistent with interim results from DANTE Trial (20), which demonstrated that the patients with PD‐L1 CPS ≥10 had clear higher pathologic regression benefit from the ICI, atezolizumab.

There are some limitations for this study and those includes the single arm design and being a single institution trial. In addition, the data analyses were not based on the intention‐to‐treat population (ITT), therefore, the results and data, especially the efficacy should be cautiously interpreted. A large, randomized phase II study with the similar concept and setting, KEYNOTE‐585 (study of the neoadjuvant or adjuvant chemotherapy [cisplatin + 5 FU or capecitabine] ± pembrolizumab in patients with gastric or GEJ adenocarcinoma, NCT03221426), is on‐going, which will likely confirm and validate the results.^18^ In addition, compared to the study chemotherapy regimen of KEYNOTE‐585, the combination of mFOLFOX instead of cisplatin + 5‐FU/capecitabine with pembrolizumab may better fit in the clinical practice in the United States when the benefit of the concept is validated.^19^

In conclusion, the pembrolizumab and mFOLFOX combination as perioperative therapy in patients with locally advanced adenocarcinoma of distal esophagus, GEJ, and stomach is effective with very encouraging results including favorable pathologic response and impressive long‐term survival rate. Preoperative ICI + chemotherapy increase immune response and enhances adjuvant treatment benefits and thereafter the overall DFS and OS. Therefore, the preoperative ICI + chemotherapy should be considered as the standard with further confirmation.

## AUTHOR CONTRIBUTIONS

**Weijing Sun:** Conceptualization (lead); data curation (lead); formal analysis (lead); funding acquisition (lead); investigation (lead); methodology (lead); project administration (equal); resources (equal); supervision (lead); validation (lead); visualization (lead); writing – original draft (lead); writing – review and editing (lead). **Nirmal Veeramachaneni:** Conceptualization (supporting); data curation (equal); investigation (equal); methodology (supporting); resources (lead); writing – review and editing (equal). **Raed Al‐Rajabi:** Conceptualization (equal); data curation (equal); investigation (equal); writing – review and editing (equal). **Rashna Madan:** Conceptualization (supporting); data curation (equal); formal analysis (equal); methodology (lead); validation (equal); writing – original draft (equal); writing – review and editing (equal). **Anup Kasi:** Conceptualization (equal); data curation (supporting); investigation (equal); writing – review and editing (equal). **Mazin Al‐Kasspooles:** Conceptualization (supporting); investigation (equal); writing – review and editing (supporting). **Joaquina Baranda:** Conceptualization (supporting); data curation (equal); investigation (equal); visualization (equal); writing – review and editing (equal). **Anwaar Saeed:** Conceptualization (supporting); investigation (equal); writing – review and editing (supporting). **Milind A. Phadnis:** Methodology (lead); resources (equal); software (lead); validation (lead); writing – original draft (supporting); writing – review and editing (equal). **Andrew K. Godwin:** Conceptualization (supporting); data curation (supporting); investigation (supporting); validation (supporting); writing – review and editing (supporting). **Mojtaba Olyaee:** Data curation (supporting); investigation (supporting); writing – review and editing (supporting). **Natalie Streeter:** Funding acquisition (supporting); project administration (equal); writing – review and editing (supporting). **Alykhan Nagji:** Data curation (supporting); investigation (supporting); writing – review and editing (supporting). **Junqiang Dai:** Methodology (equal); software (equal); validation (equal); writing – review and editing (supporting). **Stephen Williamson:** Conceptualization (supporting); data curation (supporting); investigation (supporting); writing – review and editing (supporting).

## FUNDING INFORMATION

Funded by Merck.

## CONFLICT OF INTEREST STATEMENT

The study sponsored by Merck with research fund. Weijing Sun, the coordinating author, is the PI of the study. Weijing Sun and Anwaar Saeed were involved in the advising board of Merck.

## Data Availability

Not applicable.
